# Looking for a Simplified Diagnostic Model to Identify Potentially Lethal Cases of Prostate Cancer at Initial Diagnosis: An ImGO Pilot Study

**DOI:** 10.3390/cancers14061542

**Published:** 2022-03-17

**Authors:** Serena Macrini, Simona Francesconi, Cecilia Caprera, Daniela Lancia, Matteo Corsi, Marco Gunnellini, Andrea Rocchi, Anjuta Pireddu, Fiovo Marziani, Claudia Mosillo, Maria Letizia Calandrella, Claudia Caserta, Diana Giannarelli, Annalisa Guida, Stefano Ascani, Sergio Bracarda

**Affiliations:** 1 Medical and Translational Oncology Unit, Department of Oncology, Azienda Ospedaliera Santa Maria, 05100 Terni, Italy; s.macrini@aospterni.it (S.M.); c.mosillo@aospterni.it (C.M.); m.calandrella@aospterni.it (M.L.C.); c.caserta@aospterni.it (C.C.); a.guida@aospterni.it (A.G.); 2 Pathology Unit, Azienda Ospedaliera Santa Maria Terni, University of Perugia, 06129 Terni, Italy; s.francesconi@aospterni.it (S.F.); c.caprera@aospterni.it (C.C.); d.lancia@aospterni.it (D.L.); m.corsi@aospterni.it (M.C.); s.ascani@aospterni.it (S.A.); 3 Medical Oncology Unit, Department of Oncology, Gubbio-Gualdo Tadino Hospital, 06024 Branca, Italy; marco.gunnellini@uslumbria1.it; 4 Medical Oncology Unit, Department of Medicine, San Giovanni Battista Hospital, 06034 Foligno, Italy; andrea.rocchi@uslumbria2.it; 5 Division of Pathology, Città di Castello Hospital, 06012 Città di Castello, Italy; anjuta.pireddu@uslumbria1.it; 6 Pathology Unit, Department of Clinical Pathology, San Giovanni Battista Hospital, 06034 Foligno, Italy; fiovo.marziani@uslumbria2.it; 7 Biostatistical Unit, Regina Elena National Cancer Institute, IRCCS, 00168 Rome, Italy; diana.giannarelli@ifo.gov.it

**Keywords:** prostate cancer, metastatic castration-resistant prostate cancer, metastatic castration-sensitive prostate cancer, cribriform pattern, intraductal carcinoma, phenotypic markers

## Abstract

**Simple Summary:**

Several genetic anomalies are recurrent in prostate cancer (PC) and allow this disease to be classified into distinct molecular subtypes; however, these anomalies have no predictive role and have limited relevance for clinical practice. Within this pilot study, metastatic castration-resistant PC (mCRPC) and metastatic castration-sensitive PC (mCSPC) were used for identifying and understanding lethal prostate tumor clones, from a genomic point of view. Some elements, such as a high Gleason Score and the presence of a cribriform pattern or intraductal carcinoma were evaluated as phenotypic markers of potentially lethal PC. Our results provide hypothesis-generating data, as the idea of evaluating mCSPC and mCRPC as a phenotypic/biologic model able to be translated in clinical practice. The confirmation of a high incidence of TP53 and BRCA2 mutations in larger trials may find a therapeutic implication through the choice of whether or not to use “more” therapy in respect to “selective” treatments approaches.

**Abstract:**

The recurrent genetic anomalies used to classify prostate cancer (PC) into distinct molecular subtypes have limited relevance for clinical practice. In consideration of WHO 2016 histological classification, which includes the introduction of Gleason Score 4 for patients with cribriform component and the definition of intraductal carcinoma as a new entity, a retrospective pilot study was conducted to investigate, by histological review, if there were any variations of Gleason Score and the incidence of intraductal carcinoma and cribriform pattern, intended as “phenotypic” markers of potentially lethal PC, among metastatic castration-sensitive PC (mCSPC) and metastatic castration-resistant PC (mCRPC) samples. Potentially predictive factors were also assessed. Among 125 cases, a variation in the Gleason Score was reported in 26% of cases. A cribriform (36%) or intraductal (2%) pattern was reported in a higher percentage. Of them, a primary Gleason pattern 4 was reported in 80% of cases. All patients with intraductal carcinoma present a *BRCA2* mutation, also found in 80% of cases with a cribriform pattern. This pilot study documented some hypothesis-generating data, as the evaluation of de novo mCSPC and mCRPC as phenotypic/biologic model to be translated in clinical practice. A cribriform pattern/intraductal carcinoma might be a marker of potentially lethal PC. The high incidence of *TP53* and *BRCA2* mutations in de novo mCSPC may also have a therapeutic implication.

## 1. Introduction

Cancer is a heterogeneous and dynamic disease characterized by a non-uniform development of genetically distinct subpopulations of tumor cells. Heterogeneity exists at interpatient, inter- and intra-tumor levels, and evolves over time [1]. Assessing tumor genetic heterogeneity may be helpful to understand the diseases’ behavior and the acquisition of resistance to oncological treatments [2,3,4,5].

Prostate cancer is a paradigmatic model for histological, clinical and molecular tumor heterogeneity. Indeed, prostate cancer is an umbrella term encompassing glandular, cribriform, trabecular, solid and unicellular tumor patterns [1]. It comprises indolent tumors, almost completely harmless, as well as aggressive tumors already presenting with a metastatic disease at initial diagnosis (de novo metastatic castration-sensitive prostate cancer [mCSPC]) or rapidly evolving to a metastatic castration-resistant status (metastatic castration-resistant prostate cancer [mCRPC]) [6,7].

This wide heterogeneity of prostate cancer slowed the development of a modern biological classification of the disease. Prostate-specific antigen (PSA), Gleason Score and tumor volume still represent the main parameters for disease prognostication [8,9]. However, several genetic anomalies are highly recurrent in prostate cancer and may classify this disease into distinct molecular subtypes. Nevertheless, these anomalies do not have a recognized predictive role and have limited relevance for clinical practice [10]. Additional research in this setting is necessary, based on histological and molecular data.

We hypothesize that mCSPC and mCRPC, known to be both heterogeneous tumors associated with a poor prognosis, may also be considered as interesting clinical models to identify and better understand lethal prostate tumor clones [11].

In consideration of the WHO 2016 histological classification, which includes the introduction of Gleason Score 4 for patients with cribriform component and the definition of intraductal carcinoma as new entity, a retrospective two-step pilot study was conducted. The first step assessed, by a histological review if there were any variations of Gleason Score and the incidence of intraductal carcinoma and cribriform pattern, intended as “phenotypic” markers of potentially lethal PC, among our mCSPC and mCRPC samples.

A second, exploratory step, limited only to these cases, aimed to search for potentially predictive factors, such as *PTEN*, *TP53*, *RB1* and *BRCA1/2*, potentially useful for a modern decision-making process in clinical practice.

## 2. Patients and Methods

### 2.1. Study Setting and Design

This was a retrospective, multicentric pilot study conducted at three oncology centers in the Umbria region in Italy (Azienda Ospedaliera Santa Maria, Terni; San Giovanni Battista Hospital, Foligno; Città di Castello and Branca Hospitals). The medical records of consecutive patients with a histologically confirmed prostate cancer diagnosed and followed at the centers mentioned above from January 2007 to July 2020, and with available histological material collected at the initial diagnosis, were reviewed for the study.

Cases matching the following inclusion criteria were divided into three groups: Group 1, patients with mCRPC; Group 2, patients with de novo, mCSPC. The third group of patients with low-risk localized disease and a Gleason Score 6 (3 + 3) served as a control; in the case of a Gleason grade upgrade under the central revision, cases will be excluded from this group.

The study has been approved by the Regional Ethical Committee (register number: 3770/19); all the patients signed informed consent to use their data for research purposes.

### 2.2. Study Objectives

The primary objective of the first step of the study was to perform a centralized, double-blind histological review of the entire study population, according to the new WHO classification [12], and define the prevalence of the cribriform pattern and intraductal carcinoma. As a secondary objective, we analyzed the treatment pattern and clinical course of the identified cases with and without cribriform and/or intraductal disease patterns.

In the second step of the study, we performed an explorative molecular analysis of a limited number of potentially predictive factors by next-generation sequencing (NGS) in some patients with cribriform and intraductal carcinoma.

### 2.3. Study Procedures

#### 2.3.1. Histological Analysis

Histological specimens stained with hematoxylin and eosin derived from initial prostate biopsies or radical prostatectomy specimens were centrally reviewed in a blinded fashion by two pathologists (SF and DL) for grade assignment and assessment of the presence of cribriform pattern and/or intraductal carcinoma. No histological material from metastatic sites was analyzed. All histological samples have been blindly reviewed by two pathologists, with any differences resolved by a third one, on the basis of the new WHO 2016 classification. Disagreements with the original diagnosis may derive from the centralized review or from differences between classifications. Due to the retrospective nature of this study, these differences had no clinical implications.

#### 2.3.2. Exploratory NGS Analysis

Six to eight sections (6–8 µm each) were used for NGS analysis. DNA for the NGS analysis was extracted using the automated MagCore Super (Diatech Pharmacogenetics, Jesi, Italy) platform from formalin-fixed and paraffin-embedded tissue specimens microdissected after the identification of the neoplastic area of interest (>50% tumor cells). The concentration and the degree of fragmentation of extracted DNA were assessed by RT-PCR (Rotor-Gene Q, Qiagen, Hilden, Germany). Gene panels were then amplified using Multiplex-PCR (Kit Myriapod NGS-LT 56G Onco Panel 9, Figure S1) and the Kit Myriapod NGS-LT BRCA 1–2 Panel, Diatech Pharmacogenetics), using 25 ng of total DNA. After purification and indexing, amplified fragments were selected by enrichment PCR. Then, libraries for the NGS (GeneStudio S5 System (Applied Biosystems by Thermo Fisher Scientific, Waltham, MA, USA) were prepared and quantified by Qubit 4.0 Fluorometer (Invitrogen by Thermo Fisher). Emulsion-PCR was performed by Ion One Touch 2 System (Life Technologies by Thermo Fisher Scientific), and the sequencing was performed by Ion S5 System platform (Applied Biosystems by Thermo Fisher Scientific), loaded with Chip Ion 530 (Applied Biosystems by Thermo Fisher Scientific). Two different panels were used to analyze the hot spot mutations in the 56 identified genes (Myriapod NGS-LT 56G Onco Panel, Diatech Pharmacogenetics) and the specific analysis of *BRCA1* and *BRCA2* (Myriapod NGS-LT BRCA 1–2 Panel, Diatech Pharmacogenetics). 

Data analysis, including the alignment to the reference human genome hg19, and the variant calling, was performed using a Myriapod NGS Workstation (Diatech Pharmacogenetics) equipped with the Myriapod NGS Data Analysis, Software (Diatech Pharmacogenetics). Filtered variants were annotated in the NCBI RefSeq database, and alignments were verified using Integrative Genomics Viewer.

### 2.4. Statistical Analysis

Data were analyzed by descriptive statistics. The association between different variables and patients’ characteristics was assessed as appropriate by the chi-square test or the Mann–Whitney test. Progression-free survival (PFS; defined as the time from the initiation of treatment to documented tumor progression, death for any cause, or lost-to-follow-up, whichever occurred first) and overall survival (OS; defined as the time from the initiation of treatment to death for any cause or lost-to-follow-up, whichever occurred first) were calculated by Kaplan–Meier analysis, differences were assessed by the log-rank test. Response to treatment was evaluated according to the RECIST 1.1. criteria. *p* < 0.05 was considered statistically significant. All analyses were performed using IBM-SPSS vers. 21.0 (IBM Corp. Released 2012. IBM SPSS Statistics for Windows, Version 21.0; IBM Corp.: Armonk, NY, USA).

## 3. Results

### 3.1. Study Population

Overall, 125 cases were identified for this analysis and underwent histological review (92 prostate biopsies and 33 radical prostatectomy specimens): of these, 50 cases were included in Group 1, 25 in Group 2, and 50 were classified as controls (Group 3). Patients’ characteristics at diagnosis are reported in Table 1.

Overall, no differences in age at diagnosis were disclosed among groups, while the PSA value in patients with de novo metastatic disease was significantly higher, with a median value of 840 ng/mL.

No difference for the distribution of metastatic sites between patients of Groups 1 and 2 was shown (Table 1). Four patients of the control group (8%) developed a metastatic disease (see thereafter), two with involvement of both bone and lymph nodes and one each with bone metastases only and lymph node disease only. No cases with visceral metastases were reported.

### 3.2. Histological Review

#### 3.2.1. Gleason Score Review

Overall, a change in Gleason scores according to the 2016 WHO classification was documented in 33 patients (26%) (Table 2). Of them, 14 presented a cribriform pattern (31% of patients with cribriform pattern). Ten of these cases (30%) were from radical prostatectomy specimens.

Sample review resulted in a 9% increase in the prevalence of pattern 4 in cases with Gleason Score 3 + 4; 4 + 3; 4 + 4 and in a 6% reduction in the case of Gleason Score 6 (Table 2).

Overall, 19 cases in Group 1 (38%) and seven cases in Group 2 (28%) had a modification of Gleason Score at centralized blinded review. In comparison, seven control group cases (14%) received a Gleason score upgrade from 6 to 7 (3 + 4 in six cases). Among these, all four cases developed metastatic disease.

#### 3.2.2. Prevalence of Cribriform Pattern and Intraductal Disease

Overall, a cribriform pattern was reported in 45 patients (36%), 28/50 (56%) in Group 1 (mCRPC), and 17/25 (68%) in Group 2 (mCSPC); as awaited in our model, no cases were found in the control group (43 cases). The majority of the cases of Group 1 with a cribriform pattern (16/28, 57.2%) presented a Gleason Score 4 + 4 = 8 and a significantly higher PSA at diagnosis than those without a cribriform pattern (*p* = 0.045), data were not found in Group 2. Only three patients (2%) with intraductal carcinoma were identified, and two of them also showed a cribriform pattern (one in Group 1 and one in Group 2), the only one presenting with intraductal carcinoma alone was found in Group 2 (Table 3).

Patients with cribriform patterns differed significantly in their Gleason Score compared to those without this pattern (*p* = 0.02) (Table S1), but not for age (*p* = 0.66) or PSA at diagnosis (*p* = 0.98). Detailed characteristics of cases with a cribriform pattern are described in Table 4.

Patients without a cribriform pattern had a regular distribution in their Gleason Scores, while 36/45 patients (80%) with a cribriform pattern had a primary Gleason pattern of 4 (*p* = 0.02) (Table S1).

**Figure 1:**
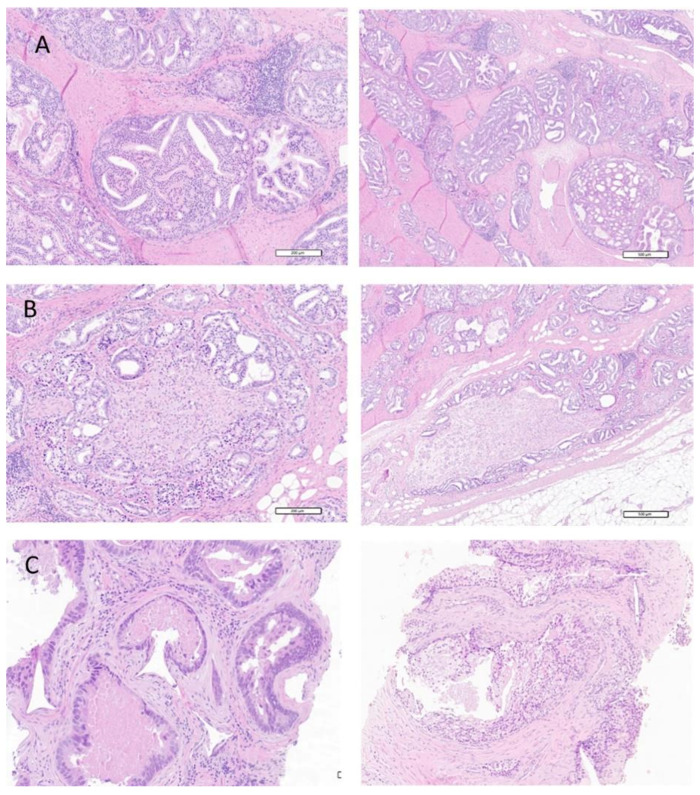
Selected histological images of patients with cribriform or intraductal carcinoma. (**A**) Histological images of the most representative cases of prostate cancer with a cribriform pattern: medium and large glands with irregular contours, jagged edges, with irregularly and fissured lumen separated into compartments by cell bridges without interposed stroma. (**B**) Prostate cancer with cribriform pattern and neuroinvasion of a ganglion: perineural invasion defined as cancer cell invasion around and through nerves. (**C**) Intraductal carcinoma of the prostate: large glands, comedonecrosis characterized by intraluminal necrotic cells, significant nuclear atypia including enlargement, anisonucleosis, prominent nucleoli and preservation of basal cells around these glands. Scale bar = 200 µm.

Of interest, the prevalence of cribriform components in cases with a Gleason Pattern 4 is significantly higher in Group 2 (de novo mCSPC) than in Group 1 (mCRPC) (Table S1, 70% versus 30%, *p* = 0.001), suggesting a potential higher aggressiveness in these cases. Selected histological images of patients with cribriform or intraductal carcinoma are displayed in Figure 1.

### 3.3. Secondary Analyses

#### 3.3.1. Patterns of Treatment and Clinical Outcomes

The median follow-up for Groups 1 and 2 was 45.0 months (range: 2–158), with 52.5 months in group 1 (range: 7.6–158.3) and 22.0 months in Group 2 (range: 1.8–115.3).

Treatment patterns and clinical outcomes for patients of Groups 1 and 2, with and without cribriform patterns, who received a first-line therapy (standard therapies or clinical trials) are reported in Table 5. In total, 21 patients (84%) in Group 2 (mCSPC) received androgen-deprivation therapy plus early chemotherapy with docetaxel. Among these cases, 16 (76.1%) patients had a cribriform pattern. The objective response rate in patients of this group with measurable disease was 40% in patients without a cribriform pattern and 100% in the five patients with a cribriform pattern (*p* < 0.03) (Table S2).

PFS after early docetaxel chemotherapy was shorter in patients with a cribriform pattern than in those without (11.2 months vs. 22.9 months; HR: 1.45; 95% CI: 0.38–5.51; *p* < 0.584). The limited number of cases evaluated in these groups does not allow a more detailed statistical analysis to be performed. Among the remaining 54 patients of Groups 1 and 2, no significant differences were disclosed in terms of best response to first-line therapy (*p* = 0.38), the number of prior lines of treatment (*p* = 0.78), PFS rates (*p* = 0.58), and OS (*p* = 0.61) (Tables S2 and S3).

Considering Groups 1 and 2 as a whole, a non-significant trend towards a lower 3-year OS rate was observed in patients with a cribriform pattern, compared with those without (68.3% vs. 80.2%, *p* = 0.61).

#### 3.3.2. Exploratory NGS Data

**Figure 2:**
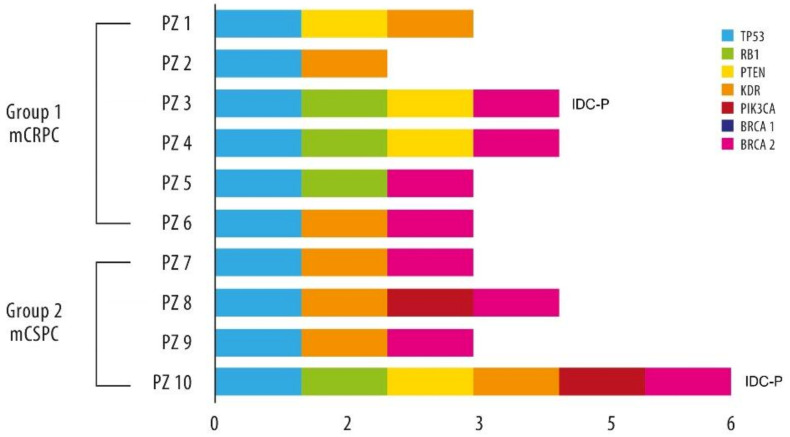
Patterns of mutation in the 10 patients with exploratory NGS data. IDC-P = intraductal carcinoma of prostate.

Results of the NGS analysis are available for only 10 cases (four in Group 1 and six in Group 2, diagnosed from January 2012 to July 2020) out of 46 patients with a cribriform pattern and intraductal component. Cases were chosen randomly, regardless of the percentage of pattern presentation. Mutation details for each patient are reported in Figure 2, while Table S4 shows all the observed mutations in the 58 genes analyzed.

Mutations in the *TP53* gene were identified in all analyzed cases in exon 4, codon c.215C > G (allelic frequencies > 99%). Seven patients showed mutations in *KDR* (exon 7, codon c.889G > A and/or exon 11, codon c.1416A > T; allelic frequencies of approximately 45%). Four cases had a mutation in *RB1* and four in *PTEN*, in both cases three out of four belong to group 2. Two mutations of the *PIK3CA* gene in Group 2 were also observed. No mutations of *ATM* and *AKT1* were found, contrary to what was expected in the literature.

Data of the separate analysis for *BRCA1* and *BRCA2* genes are available for the same ten patients (Table S5). A codon7007G > A mutation in different exons of the *BRCA2* gene was found in eight of the ten tested cases (80%), all with a cribriform pattern and two with an intraductal component also. Two patients resulted in wild-type for both *BRCA1* and *BRCA2*.

## 4. Discussion

This retrospective pilot study was designed to explore new potential prognostic and predictive factors of potential use for modern daily decision-making in prostate cancer.

Our results showed a relevant variation in the Gleason Score (26% of cases) in the review of archival tumor tissue according to the 2016 WHO classification [12]. An overall reduction of 3 + 3 Gleason Score pattern was reported (6% of cases), along with an increase of primary Gleason pattern 4 in 9% of cases, in line with literature data [13,14]. This is mainly related to substantial changes concerning the inclusion in the Gleason pattern 4 of malformed glands without significant cytological atypia, previously [15] classified as a Gleason pattern 3.

It is now well established that a tumor with a Gleason Score 6 has a better prognosis than a tumor with a Gleason Score 7. In line with this observation, four out of seven patients of our control group who developed a metastatic disease had an increase in their Gleason Score after review, further confirming the validity of the new classification system.

The cribriform growth pattern is one of the histological factors more frequently associated with a negative prognosis. The histological review led to identifying the presence of a cribriform (36%) or intraductal (2%) pattern in a higher percentage of reviewed cases for the study. Of note, two out of three intraductal cases are also associated with a cribriform pattern. As a confirmation, none of the patients reviewed in the control group presented a cribriform or intraductal disease.

Prostate cancer patients with cribriform/intraductal patterns present a primary Gleason pattern 4 in 80% of cases. Moreover, these patients are mainly represented in the highly aggressive mCSPC Group 2, confirming the negative prognostic role of this pattern and providing a rationale for the well-known aggressiveness of the disease in this setting [16,17,18].

In total, 21 of 25 patients of Group 2 (mCSPC) received early docetaxel chemotherapy. In this group, patients with the cribriform component appear to have a better response rate and a lower median PFS than the other cases (median PFS 11.2 months in the cribriform group vs. 22.9 months in the patients without cribriform pattern). Of course, these data should be considered in light of the overall low number of cases of our pilot study, but the potential role of a cribriform and/or intraductal pattern not only as negative prognostic factors but also as a phenotypic marker of potentially lethal tumoral clones in patients with de novo mCSPC may be considered and needs confirmation in larger trials on well-selected patients.

The high percentage of patients found with a somatic *BRCA2* mutation underlying an aggressive phenotype is worth attention. In patients with intraductal carcinoma, the incidence of *BRCA2* germline mutations reported in the literature is higher than for adenocarcinoma, while the data for somatic mutations are similar to the rest of patients with prostatic carcinoma [19,20]. In our study, both the tested patients with intraductal carcinoma present a mutation in *BRCA2* also found in 80% of the NGS tested cases with a cribriform pattern. Again, these data should be considered hypothesis-generating to be verified in larger series of patients with cribriform growth patterns and intraductal carcinoma.

Furthermore, our exploratory data shows an increased proportion of patients with *TP53* mutation compared with the literature, approximately 30% [21,22], and the co-presence of *TP53* and *RB1* genes mutations in four out of 10 cases. In contrast, in the literature, a biallelic loss of *TP53* and *RB1* is reported in only 4% of the cases [23]. Notably, the frequency of the combination of *TP53* loss and *RB1* loss approaches 100% in tumors with a small-cell/neuroendocrine component, characterized by a well-known clinical aggressiveness and generally not responding to the androgen receptor (AR)-directed therapy [21,24].

Of note, a combination of *TP53*, *RB1* and *PTEN* mutations was reported in the two patients showing both cribriform patterns and intraductal carcinoma. Aberrations in these three genes are key drivers of therapy resistance in prostate cancer and associated with poorer overall survival, while the incidence of loss of cytoplasmic PTEN protein is doubled in intraductal carcinomas with fair 84% compared to acinar carcinoma, estimated between 9% and 40% [25,26,27]. Of note, no *ATM* and *AKT1* gene mutations were observed in our cases, differing from available studies [22,28].

Our pilot study presents major limitations, mainly the low number of cases, a retrospective study design, and the limited number of genomic evaluations, which should be considered only exploratory data.

## 5. Conclusions

This pilot study documented some intriguing and hypothesis-generating data, such as the idea of evaluating de novo mCSPC and mCRPC as in vivo models of aggressiveness to define a realistic phenotypic/biologic model able to be translated in daily clinical practice and not limited to purely research scenarios. We suggest using the presence of a cribriform pattern/intraductal carcinoma as a preliminary useful “phenotypic” marker of potentially lethal prostate cancer. Furthermore, the high incidence of mutations in the *TP53* and *BRCA2* genes in de novo mCSPC, if confirmed in larger trials, may have a clinical therapeutic implication through the choice of whether or not to use “more” therapy options in this aggressive setting (e.g., adding chemotherapy or new hormone agents) in respect to use “selective” treatments approaches: more is not always better.

## Figures and Tables

**Table 1:** Baseline patients’ characteristics.

	Group 1: mCRPC (*n* = 50)	Group 2: mCSPC (*n* = 25)	Group 3: Control (*n* = 50)	Total (*n* = 125)
Age (years), median (range)	69 (55–84)	67 (49–83)	70 (52–84)	69 (49–84)
PSA value at diagnosis (ng/mL), median (range)	46 (1.7–419.0)	843 (13.2–5800)	7 (2.0–35.0)	177 (1.67–5800)
Initial Gleason Score, *n* (%):				
Confirmed	31 (62)	18 (72)	43 (86)	92 (74)
Modified	19 (38)	7 (28)	7 (14)	33 (26)
Lymph node metastases, *n* (%):				
No	17 (34)	4 (16)	48 (96)	69 (55.2)
Yes	33 (66)	21 (84)	3 (6)	56 (44.8)
Bone metastases, *n* (%):				
− No	20 (40)	2 (8)	47 (94)	69 (55)
− Yes	30 (60)	23 (92)	3 (6)	56 (45)
Visceral metastases, *n* (%):				
− Lung	4 (8)	4 (16)	0	8 (6)
− Liver	1 (2)	2 (8)	0	3 (2)
− Other	1 (2)	2 (8)	0	3 (2)

**Table 2:** Variations in Gleason Score.

Gleason Score	Group 1: mCRPC (*n* = 50), *n* (%)		Group 2: mCSPC (*n* = 25), *n* (%)		Group 3: Control (*n* = 50), *n* (%)	
	Initial	After Revision	Initial	After Revision	Initial	After Revision
3 + 3	0	1 (2)	1 (4)	1 (4)	50 (100)	43 (86)
3 + 4	13 (26)	7 (14)	1 (4)	1 (4)	0	6 (12)
4 + 3	9 (18)	7 (14)	2 (8)	5 (20)	0	1 (2)
4 + 4	14 (28)	21 (42)	7 (28)	8 (32)	0	0
4 + 5	6 (12)	11 (22)	7 (28)	6 (24)	0	0
5 + 3	5 (10)	0	2 (8)	0	0	0
5 + 4	3 (6)	3 (6)	3 (12)	3 (12)	0	0
5 + 5	0	0	2 (8)	1 (4)	0	0

**Table 3:** Prevalence of cribriform pattern and intraductal carcinoma in the histologically reviewed population.

	Group 1: mCRPC (*n* = 50), *n* (%)	Group 2: mCSPC (*n* = 25), *n* (%)	Group 3: Control (*n* = 43), *n* (%)	Total (*n* = 118), *n* (%)
Cribiform pattern:				
Present	28 (56)	17 (68)	0	45 (38)
Absent	22 (44)	8 (32)	43 (100)	80 (62)
Intraductal carcinoma:				
Present	1 (2)	2 (8)	0	3 (3)
Absent	49 (98)	23 (92)	43 (100)	115 (97)

**Table 4:** Characteristics of patients with a cribriform pattern.

	Group 1: mCRPC (*n* = 28), *n* (%)	GROUP 2: mCSPC (*n* = 17), *n* (%)	Total (*n* = 45), *n* (%)
Gleason score after revision:			
− 3 + 3	0	0	0
− 3 + 4	3 (11)	1 (6)	4 (9)
− 4 + 3	3 (11)	3 (17)	6 (13)
− 4 + 4	16 (56)	7 (41)	23 (50)
− 4 + 5	3 (11)	4 (24)	7 (16)
− 5 + 4	3 (11)	1 (6)	4 (9)
− 5 + 5	0	1 (6)	1 (3)
Intraductal carcinoma:			
− Present	1 (4)	1 (6)	2 (5)
− Absent	27 (96)	16 (94)	43 (95)
Age (years), median (range)	68 (57–84)	66 (49–78)	67 (49–84)
PSA value at diagnosis, median (range)	69 ng/mL (2–419)	726 ng/mL (13–3500)	300 ng/mL (1.7–3500)
Lymph node metastases:			
− Yes	19 (68)	14 (83)	33 (73)
− No	9 (32)	3 (17)	12 (27)
bone metastases:			
− Yes	15 (54)	16 (94)	31 (69)
− No	13 (46)	1 (6)	14 (31)
− Number of lesions, median (range)	4 (1–10)	5 (1–21)	
Visceral metastases:			
− Lung	2 (7)	3 (18)	5 (11)
− Liver	0	2 (12)	2 (4)
− Other	0	1 (6)	1 (2)

**Table 5:** Treatment and clinical outcomes in patients with and without a cribriform pattern in the histologically reviewed population.

	Group 1: mCRPC (*n* = 50), *n* (%)	Group 2: mCSPC (*n* = 25), *n* (%)	Group 3: control (*n* = 43), *n* (%)
Early docetaxel chemotherapy	0	21 (84)	0
mCRPC first-line treatment:			
− Yes	42 (84)	14 (56)	0 (0)
− No	8 (16)	11 (44)	43 (100)
mCRPC second-line treatment:			
− Yes	20 (40)	8 (32)	0
− No	30 (60)	17 (68)	43 (100)
mCRPC third-line treatment:			
− Yes	8 (16)	2 (8)	0
− No	42 (84)	23 (92)	43 (100)
mCRPC other treatment:			
− Yes (range 4–6)	4 (8)	0	0
− No	46 (92)	25 (100)	43 (100)

## Data Availability

Data may be made available upon reasonable request.

## Supplementary Materials

The following are available online at https://www.mdpi.com/article/10.3390/cancers14061542/s1, Figure S1: Genes included in the Multiplex-PCR amplification, Table S1: Revision of grade group in patients with and without cribiriform pattern, Table S2: Clinical outcomes and characteristics of patients of group 2 mCSPC with and without cribriform pattern, Table S3: Clinical outcomes and characteristics of patients of Group 1 mCRPC with and without cribriform pattern, Table S4: Mutations reported at the NGS analysis, Table S5: Mutations reported in BRCA1 and 2 genes.
