# Supplementary material for: Looking for a Simplified Diagnostic Model to Identify Potentially Lethal Cases of Prostate Cancer at Initial Diagnosis: An ImGO Pilot Study
Source: Cancers (Basel). 2022 Mar 17;14(6):1542. doi: 10.3390/cancers14061542 (PMC8946832; doi:10.3390/cancers14061542)
Supplement: Supplementary file 1 [file cancers-14-01542-s001.zip › cancers-1586322-supplementary.pdf]

Supplementary Tables

Table S1. Revision of grade group in patients with and without cribiriform pattern.

Grade group	Patients without cribriform pattern, n (%)	Patients with cribriform pattern, n (%)	p-value
Before revision			
1	2 (6.7)	0	
2	4 (3.3)	4 (8.9)	
3	6 (20)	6 (13.3)	*p<0.041*
4	6 (20)	23 (51.1)	
5	12 (40)	12 (26.7)	
Total	30 (100)	45 (100)	
After revision			
3	6 (27.3%)	6 (16.7%)	
4	6 (27.3%)	23 (63.9%)	*p<0.022*
5*	10 (45.4%)	7 (19.4%)	
Total	22 (100%)	36 (100%)	

* Only primary pattern 4.

Table S2. Clinical outcomes and characteristics of patients of group 2 mCSPC with and without cribriform pattern.

	Patients with cribiform pattern (n=17), n (%)	Patients without cribiform pattern (n=6), n(%)	p-value
DCT CT* mCSPC	16 (94.1)	5 (62.5)	0.048
DCT CT* tumor response:	16 (94.1)	5 (62.5)	
• CR	0	0	
• PR	6	5	0.030
• SD	9	0	
• PD	0	0	
First-line therapy mCRPC:			
• No	8 (47.1)	3 (37.5)	0.49
• Yes (abiraterone/enzalutamide/docetaxel/cabazitaxel/other)	9 (52.9)	5 (62.5)	
PFS first-line therapy (months), (95% CI)	11.2 (6.2–24.7)	22.9 (7.7–nv)	0.584
OS:			
• 1-year OS	93.1 %	100 %	
• 2-year OS	93.1 %	100 %	0.88
• 3-year OS	79.8 %	71.4 %	

DCT CT: Early docetaxel chemotherapy. mCSPC: metastatic castration-sensitive prostate cancer; mCRPC: metastatic castration-resistant prostate cancer. PFS: progression free survival; OS: overall surivival.

Table S3. Clinical outcomes and characteristics of patients of Group 1 mCRPC with and without cribriform pattern.

	Patients with cribi-form pattern (n=28), n (%)	Patients without cribiform pattern (n=22), n (%)	p-value
mCRPC first-line therapy:<br>• No<br>• Yes (abiraterone/enzalutamide/docetaxel/caba-zitaxel/other)	5 (17.9)<br>23 (82.1)	3 (13.6)<br>19 (86.4)	0.92
First-Line mCRPC tumor response:<br>• PR<br>• SD<br>• PD	23 (82)<br>2<br>4<br>13<br>4	19 (86.4)<br>0<br>4<br>12<br>1	0.68
PFS first-line therapy (months), (95% CI)	13.3 (7.6–18.12)	11.1 (4.11–21.5)	0.605
mCRPC second-line therapy:<br>• No<br>• Yes (abiraterone/enzalutamide/docetaxel/caba-zitaxel/other)	17 (60.7)<br>11 (39.3)	13 (59.1)<br>9 (40.9)	0.73
mCRPC third-line therapy:<br>• No<br>• Yes (abiraterone/enzalutamide/docetaxel/caba-zitaxel/other)	25 (89.3)<br>3 (10.7)	17 (77.3)<br>5 (22.7)	0.52
OS<br>• 1-year OS<br>• 2-year OS<br>• 3-year OS	92.6%<br>87.9%<br>63.7%	89.7%<br>89.7%<br>89.7%	0.295

mCSPC: metastatic castration-sensitive prostate cancer; mCRPC: metastatic castration-resistant prostate cancer. PFS: progression free survival; OS: overall surivival.

Table S4. Mutations reported at the NGS analysis.

Patient	Gene	Exon	c.	p.	FA (%)	Results
1	*PTEN*	–	–	–	–	Synonymous mutation (Lys144Lys)
	TP53	4	c.215C>G	p.Pro72Arg	99.73	Mutat. missense (drug response)
	KDR	11	c.1416A>T	p.Gln472His	48.33	Mutat. missense (not provided)
2	*TP53*	8	c.880G>T	p.Glu294*	43.17	Pathogenic stop gained
	KDR	11	c.1416A>T	p.Gln472His	52.32	Mutat. missense (not provided)
3	*TP53*	4	c.215C>G	p.Pro72Arg	99.19	Mutat. missense (drug response)
	RB1	23	c.2327C>T	p.Pro776Leu	99.67	Mutat. missense and splice region variant
4	*PTEN*	–	–	–	–	Synonymous mutation (Lys144Lys)
	TP53	4	c.215C>G	p.Pro72Arg	99.5	Mutat. missense (drug response)
		8	c.920-4C>T	–	66.96	Splicing region variant

	RB1	23	c.2327C>T	p.Pro776Leu	99.79	Mutat. missense and splice region variant
**5**	*PTEN*	–	–	–	–	SYNONYMOUS MUTATION (Lys144Lys)
	TP53	4	c.215C>G	p.Pro72Arg	100	Mutat. missense (drug response)
		4	c.375+5G>T	–	42.06	Likely pathogenic
	RB1	23	c.2327C>T	p.Pro776Leu	99.76	Mutat. missense and splice region variant
**6**	*TP53*	4	c.215C>G	p.Pro72Arg	98.47	Mutat. missense (drug response)
		5	c.530C>T	p.Pro177Leu	51.09	Mutat. missense uncertain sign
	KDR	11	c.1416A>T	p.Gln472His	100	Mutat. missense (not provided)
		7	c.889G>A	p.Val297Ile	48.2	Mutat. missense (not provided)
**7**	*TP53*	4	c.215C>G	p.Pro72Arg	98.95	Mutat. missense (drug response)
		8	c.920-4C>T	–	59.78	Splicing region variant
	KDR	11	c.1416A>T	p.Gln472His	47.57	Mutat. missense (not provided)
**8**	*TP53*	4	c.215C>G	p.Pro72Arg	99.76	Mutat. missense (drug response)
		4	c.375+5G>A	–	35.67	Splice region uncertain sign
		5	c.503A>T	p.His168Leu	30.29	Mutat. missense
	KDR	11	c.1416A>T	p.Gln472His	48.03	Not provided
	PIK3CA	10	c.1624G>A	p.Gln542Lys	38.38	Pathogenic/likely pathogenic
**9**	*TP53*	4	c.215C>G	p.Pro72Arg	99.84	Mutat. missense (drug response)
		7	c.754delC	p.Leu252fs	46.84	Frameshift variant
		8	c.817C>G	p.Arg273Gly	43.3	Mutat. missense
	KDR	11	c.1416A>T	p.Gln472His	42.77	Mutat. missense (not provided)
		7	c.889G>A	p.Val297Ile	41.5	Mutat. missense (not provided)
**10**	*PTEN*	–	–	–	–	Synonymous mutation (Lys144Lys)
	TP53	4	c.215C>G	p.Pro72Arg	99.77	Mutat. missense (drug response)
	RB1	23	c.2327C>T	p.Pro776Leu	99.64	Mutat. missense and splice region variant
	KDR	7	c.889G>A	p.Val297Ile	47.07	Mutat. missense (not provided)
	PIK3CA	7	c.1173A>G	p.Ile391Met	55.72	Benign

FA: allelic frequencies. Mutat.: mutation.

Table S5. Mutations reported in *BRCA1* and *2* genes.

Patients	Gene	Esone	c.	p.	FA (%)	Results
1	*BRCA1*	All WT				
	BRCA2					
2	*BRCA1*	All WT				
	BRCA2					
3	*BRCA2*	14	c.7238A>G	p.Lys2413Arg	80.84	Missense_variant
4	*BRCA2*	10	c.1646A>G	p.Lys549Arg	61.03	Missense variant
5	*BRCA2*	11	c.2578A>G	p.Ile860Val	59.64	Missense variant
			c.6434A>C	p.Asn2145Thr	77.73	
6	*BRCA2*	11	c.2578A>G	p.Ile860Val	59.64	Missense variant
7	*BRCA2*	14	c.7397T>C	p.Val2466Ala	96.03	Missense variant
8	*BRCA2*	22	c.8796C>G	p.His2932Gln	41.44	Missense variant
9	*BRCA2*	11	c.4689G>A	p.Trp1563*	12.4	Stop gained
10	*BRCA2*	22	c.8796C>G	p.His2932Gln	46.35	Missense variant
		27	c.9730G>C	p.Val3244Leu	82.69	

FA: allelic frequencies. WT: wild-type.

Figure S1. Genes included in the Multiplex-PCR amplification.

ABL1	CSF1R	FBXW7	GNAS	KIT	NPM1	SKT11
AKT1	CTNNB1	FGFR1	HNF1A	KRAS	NRAS	SMAD4
ALK	DDR2	FGFR2	HRAS	MAP2K1	PDGFRA	SMARCB1
APC	DNMT3A	FGFR3	IDH1	MET	PIK3CA	SMO
ATM	EGFR	FLT3	IDH2	MLH1	PTEN	SRC
BRAF	ERBB2	FOXL2	JAK2	MLP	PTPN11	TP53
CDH1	ERBB4	GNA11	JAK3	MSH6	RB1	TSC1
CDKN2A	EZH2	GNAQ	KDR	NOTCH	RET	VHL
